# The Cholera

**Published:** 1892-09

**Authors:** 


### THE CHOLERA.

---

Both newspapers and medical journal, for some time have been telling of the ravages of the cholera in Europe and the East. Many accounts have been highly sensational; others strictly conservative.

The present epidemic which is creating such excitement in Europe and even some apprehension in our own country, is an extension of the disease from India, where it is endemic, through Afghanistan and Persia, along the line of the Transcaspian Railway. It crossed the Russian frontier about the middle of May. In spite of the most strenuous efforts on the part of the government to check the progress of the disease by quarantine regulations, disinfection, sanitary inspection, etc., it continued to spread itself along the great lines of travel with a rapidity that was alarming. The mortality was very great, and especially so in the districts only a short time before visited by famine. Official statistics show that up to the end of June there had been 25,000 deaths in the Russian Empire.

It gradually worked its way westward, and at recent accounts had gone into Poland. Every effort was made to protect Moscow and St. Petersburg, but nothing availed. The cities became seized with panic and confusion, business was suspended, and the inhabitants fled. The nations of Northern and Western Europe are much alarmed and are adopting every precaution of protection. The Austro-Russian frontier is guarded by the military. In Hamburg "emigrants are forbidden to leave the train at the station; they are conveyed direct to the wharf, where they must remain in specially constructed pavilions until their ship leaves. At five stations near the Russian frontier an examination of railway passengers from Russia has been instituted, and temporary hospitals erected."

In July the daily average number of cases was 8,601, and the average number of deaths per day was 4,268, throughout all Russia.

The total number of new cases reported in Russia for August 22d, was 6,806; number of deaths, 3,429.

In Paris and other places in France there seems to be no doubt that the epidemic of cholerine or choleriform diarrhœa (so-called) is true cholera, and Spain has quarantined against Paris. The comma-bacillus of Koch has been found in the dejections of "cholerine" patients. This would appear to be decisive.

Latest dispatches announce the prevalence of the cholera at Hamburg and Hâvre. These parts are in constant communication by steamer with New York, Philadelphia and Baltimore, and some fear is being entertained that these cities will contract the epidemic. The last visit of Asiatic cholera to our shores was in 1873. Government officials will establish a quarantine against all European ports.

German investigators are busying themselves with the question of occulation as a means of precaution. Brieger and Wasserman have succeeded in rendering guinea-pigs immune to cholera by injections with cholera bacilli cultivated on extract of the thymus gland. Whether this discovery will develop into anything more than interesting laboratory information remains to be seen.

